# Cell Adhesion in Zebrafish Embryos Is Modulated by March8

**DOI:** 10.1371/journal.pone.0094873

**Published:** 2014-04-21

**Authors:** Mi Ha Kim, Martha L. Rebbert, Hyunju Ro, Minho Won, Igor B. Dawid

**Affiliations:** Program in Genomics of Differentiation, Eunice Kennedy Shriver National Institute of Child Health and Human Development, National Institutes of Health, Bethesda, Maryland, United States of America; NHLBI, NIH, United States of America

## Abstract

March8 is a member of a family of transmembrane E3 ubiquitin ligases that have been studied mostly for their role in the immune system. We find that March8 is expressed in the zebrafish egg and early embryo, suggesting a role in development. Both knock-down and overexpression of March8 leads to abnormal development. The phenotype of zebrafish embryos and Xenopus animal explants overexpressing March8 implicates impairment of cell adhesion as a cause of the effect. In zebrafish embryos and in cultured cells, overexpression of March8 leads to a reduction in the surface levels of E-cadherin, a major cell-cell adhesion molecule. Experiments in cell culture further show that E-cadherin can be ubiquitinated by March8. On the basis of these observations we suggest that March8 functions in the embryo to modulate the strength of cell adhesion by regulating the localization of E-cadherin.

## Introduction

Embryonic development depends on controlled cell adhesion to assure integrity of the embryo while allowing cell movements. Thus control of the abundance and localization of adhesion molecules in embryonic cells is critical for development. In frog and fish embryos, as in others, cadherins are premier adhesion factors that bear a major responsibility for regulating the shape of the embryo and the behavior of its cells [1], [2], [3]. E-cadherin, the major adhesion molecule active in zebrafish development, is encoded by the *cdh1* locus. E-cadherin is maternally expressed and essential for blastomere cleavage [4]. This factor is further indispensible for the process of epiboly, and mediates cell-cell adhesion in convergence and extension movements during gastrulation in zebrafish development [5], [6], [7], [8]. Similarly, early Xenopus embryos express cadherins, which are required for cell adhesion in the blastula, and for cell migration and morphogenesis during gastrulation [9], [10], [11], [12], [13].

Cell adhesion is subject to multiple levels of regulation [14], [15]. The abundance of cell surface proteins is controlled at many levels including intracellular localization and protein stability, and cadherins are continually turned over through internalization followed by recycling to the plasma membrane or degradation [16]. Ubiquitination by the E3 ubiquitin ligase Hakai is involved in the dynamics of cadherin localization [17].

The March (membrane-associated RING-CH) family of E3 ubiquitin ligases was discovered as representing cellular homologs of viral proteins that interfere with host defenses [18]. Most but not all the 11 members of the family share a basic structure with the founding member c-MIR, now named March8, containing an N-terminal RING finger domain and two transmembrane domains [18], [19], [20]. Function of March8 and the closely related March1 has been studied mostly in immune cells where these proteins mediate the ubiquitination and downregulation of immune regulatory cell surface molecules including MHC II, Fas, CD86 (B7.2), and others [19], [21], [22], [23], [24], [25]. March8 also controls cell surface expression of some additional proteins [26], [27]. While the function of March8 in immune system cells derived from adult organisms and other cultured mammalian cells has been studied broadly, nothing is known about its possible function in the vertebrate embryo. We observed expression of March8 in early embryos of zebrafish and Xenopus, suggesting that this protein might have a role in embryogenesis. Here we report studies using knock-down and overexpression experiments indicating that appropriate levels of March8 expression are essential for survival and maintenance of cell adhesion in the embryo, at least in part by regulating the levels of E-cadherin at the surface of embryonic cells.

## Results

### Identification of *March8* orthologs in zebrafish and Xenopus genomes

To examine the role of March8 in embryogenesis, we identified a *march8* gene in zebrafish highly similar to human *MARCH8*. The zebrafish gene and protein showed 74% nucleic acid identity and 78% amino acid identity with their human counterparts (Figure S1). Amino acid alignment and domain analysis indicate that the zebrafish protein is highly conserved with respect to functional domains characterized in other vertebrate species, including a RING domain at the N-terminus, two transmembrane domains, and a tyrosine-based motif (YXXΦ) at the C-terminus. The same is true of the Xenopus protein (Figure S1).

### *March8* expression during zebrafish development

To characterize the *march8* expression profile, we performed RT-PCR from different stage embryos. Maternal expression of *march8* was detected in cleaving embryos and decreased gradually during gastrulation (Figure S2A). Then zygotic expression of *march8* increased during somitogenesis, and expression continued at least until 48 hours post fertilization (hpf). Spatial expression patterns of *march8* were examined by whole-mount in situ hybridization on selected stages from cleavage through 2 days post fertilization (dpf). During cleavage stages, *march8* was expressed in all blastomeres, followed by a rapid decrease during gastrulation; later expression was largely restricted to the brain (Figure S2B).

### Morpholino knockdown of March8 causes apoptosis and abnormal development

**Figure 1:**
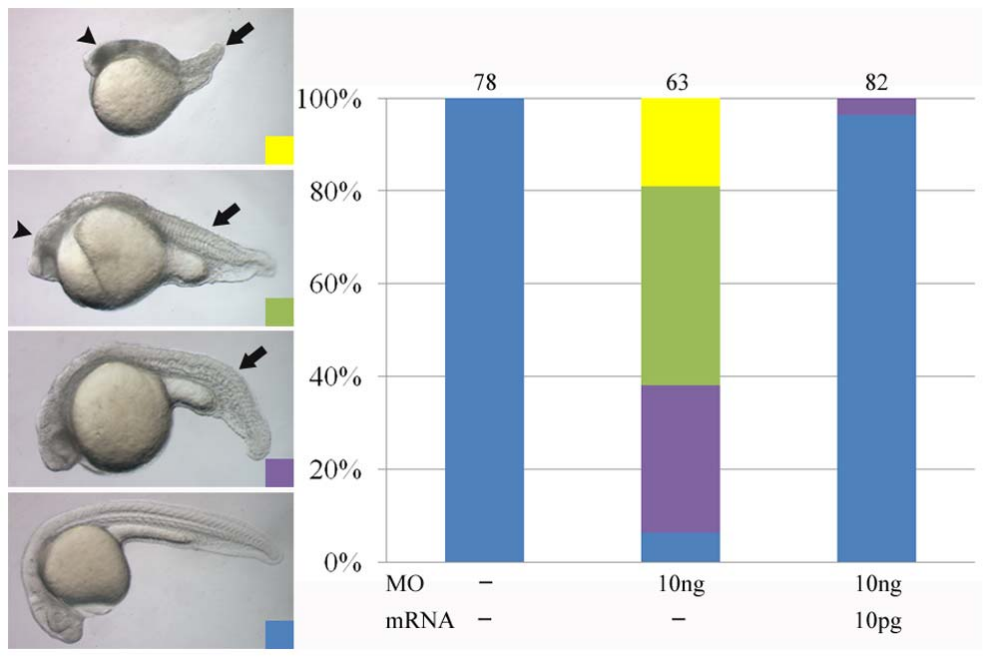
*March8* morphants show developmental defects. (A) March8 MO-injected embryos showed different levels of morphological defects as compared with WT, including cell death (arrowhead), shortened body axis and kinked tail (arrow). (B) Quantitative evaluation of defects in embryos injected with 10 ng March8 MO. These defects were rescued by coinjection with 10 pg *march8* mRNA. Number of embryos is listed on top of bars.

While March8 function in immune cells has been studied to a considerable extent [19], [20], little is known about the possible role of this or any member of the March family in embryonic development. Given that March8 is expressed in the early embryo we used morpholino oligonucleotide (MO)-mediated inhibition to ask whether this gene is required for zebrafish development. As shown in Figure 1, injection of March8 MO led to extensive abnormalities in 24 hours post fertilization (hpf) embryos. The evidence suggests that this is a specific effect of reduction of March8 expression as the effect could be rescued almost completely by coinjection of MO-resistant *march8* mRNA (Figure 1). Likewise the rescue supports the view that MO injection achieved an effective loss-of-function phenotype for March8. Inspection of MO-injected embryos under the microscope suggested that cell death was widespread in these embryos. TUNEL staining confirmed that apoptosis is greatly increased from a very low level in the controls to a high level in the MO-injected embryos (Figure S3). The cause of increased apoptosis in these embryos is not known as it is likely that target molecules exist in the embryo that have not been identified so far. At this stage we conclude that March8 is required for normal development and cell survival in zebrafish.

### March8 overexpression induces embryonic cell dissociation

**Figure 2:**
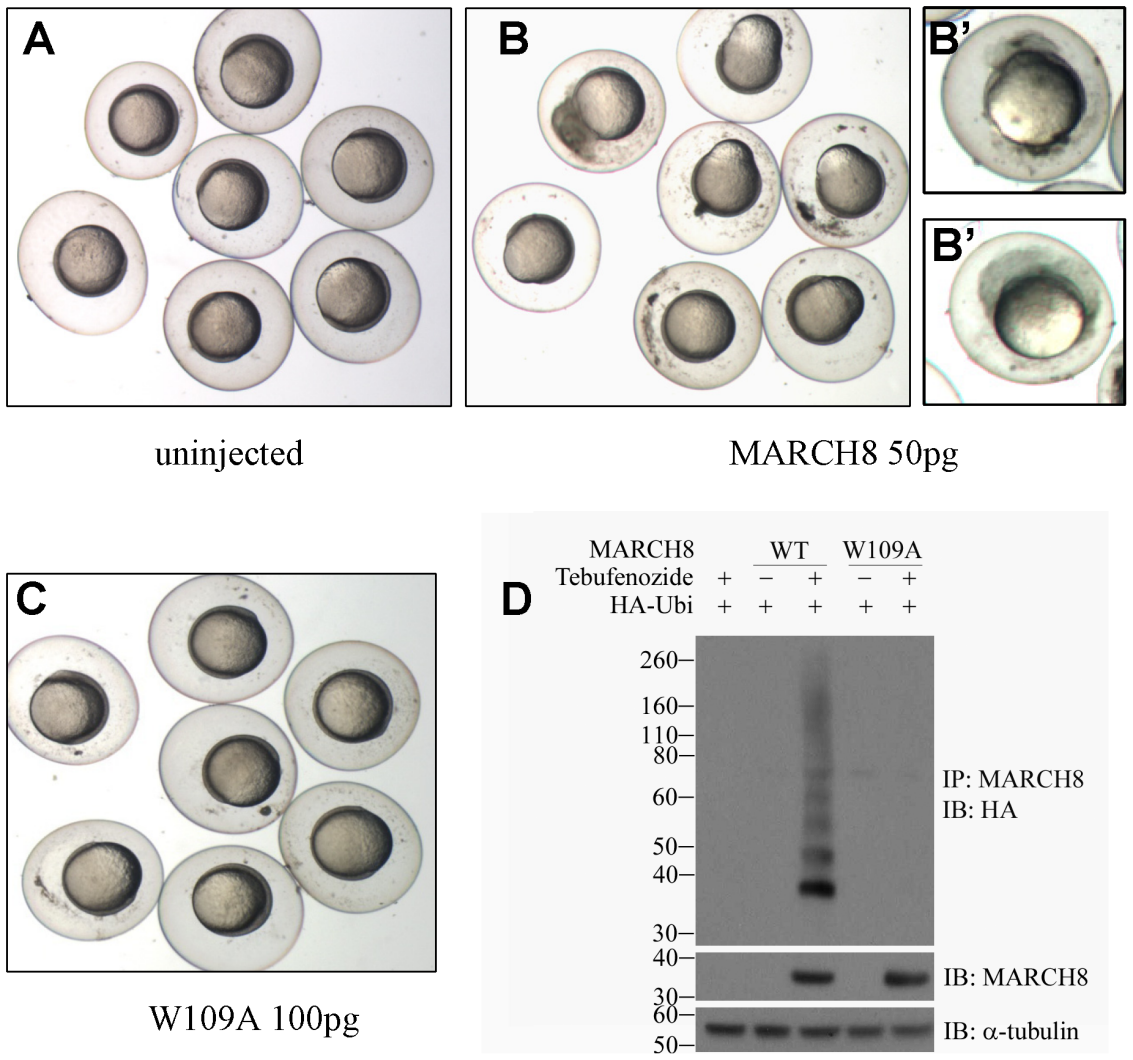
Overexpression of *march8* mRNA induces early embryonic death. (A–C) Uninjected embryos (A), and embryos injected with WT *march8* mRNA (B) or RING domain mutant (W109A) *march8* mRNA (C), at shield to 60% epiboly stage. Overexpression of March8 results in loss of cell adhesion, abnormal cell migration, and cell death (B); individual embryos showing disaggregation of embryonic cells are shown in B′. Embryos injected with W109A mutant mRNA were normal (C). (D) Self ubiquitination of March8. HEK293T cells were transfected with the plasmids indicated and activated by addition of tebufenozide (see Materials and Methods). Lysates were immunoprecipitated (IP) with anti-March8 antibody and then immunoblotted (IB) with anti-HA antibody to detect ubiquitinated proteins (upper panel). Expression of March8 and tubulin as control are shown in bottom panels.

To investigate the function of March8 during embryogenesis, we injected in vitro synthesized *march8* mRNA into 1 - 8 cell stage embryos. The first defects caused by overexpression were apparent during gastrulation, affecting epiboly movements and cell adhesion. Compared to controls (Figure 2A), the injected embryos showed aberrant epiboly (Figure 2B), and a portion of the embryos gave indication of cell dissociation (Figure 2B′). March8 is predicted to be an E3 ubiquitin ligase, and has been shown to have ligase activity [18], [19], [20]. We asked whether this enzymatic activity is required for the effect of March8 overexpression in the embryo. A conserved tryptophan residue in the RING domain of E3 ubiquitin ligases is known to be essential for binding the E2 enzyme and thus for activity [28], and thus we tested whether injection of mRNA encoding a mutant (W109A) protein would affect zebrafish embryos. As seen in Figure 2C, such embryos underwent normal gastrulation even though the dose injected was higher than that used to induce severe abnormalities with wild type (WT) mRNA. Using cultured HEK293T cells we showed that zebrafish March8 could carry out self-ubiquitination while the W109A mutant was inactive (Figure 2D), confirming that the zebrafish protein is a functional homolog of previously characterized human MARCH8 [18].

**Figure 3:**
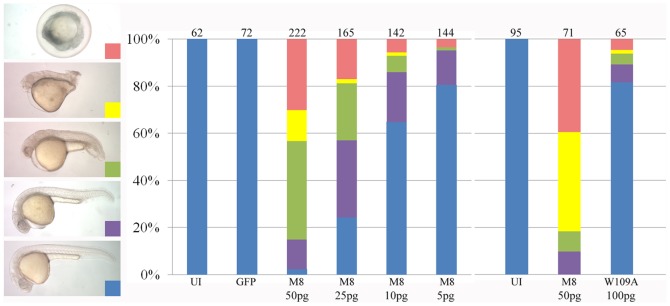
The RING domain of March8 is critical for induction of embryonic defects. The classification of morphological defects at 26 hpf after *march8* mRNA injection is shown at the left. Quantification shows that defects were induced in a dose-dependent manner by WT, but very weakly by W109A mutant *march8* mRNA. Number of embryos is listed on top of bars. UI, uninjected; GFP, embryos injected with 50 pg GFP mRNA.

When analyzed at 26 hpf, March8-overexpressing embryos showed dose dependent levels of abnormalities (Figure 3). About 30% of embryos at the 50 pg dose dissociated during gastrulation and died well before 26 hpf, and virtually all the remaining embryos exhibited morphological defects. Injection of lower doses of *march8* mRNA showed fewer and less severe defects (Figure 3). As already concluded from analysis during gastrulation (Figure 2), the enzymatic activity of March8 was required to induce abnormalities in embryos analyzed at 26 hpf: when the inactive mutant W109A was injected, over 80% of the embryos developed normally in spite of use of a high dose of mRNA (Figure 3). We conclude that overexpression of E3 ubiquitin ligase active March8 is deleterious to the embryo, leading to cell dissociation and abnormal development.

### March8 overexpression diminishes cell adhesion in Xenopus animal caps

**Figure 4:**
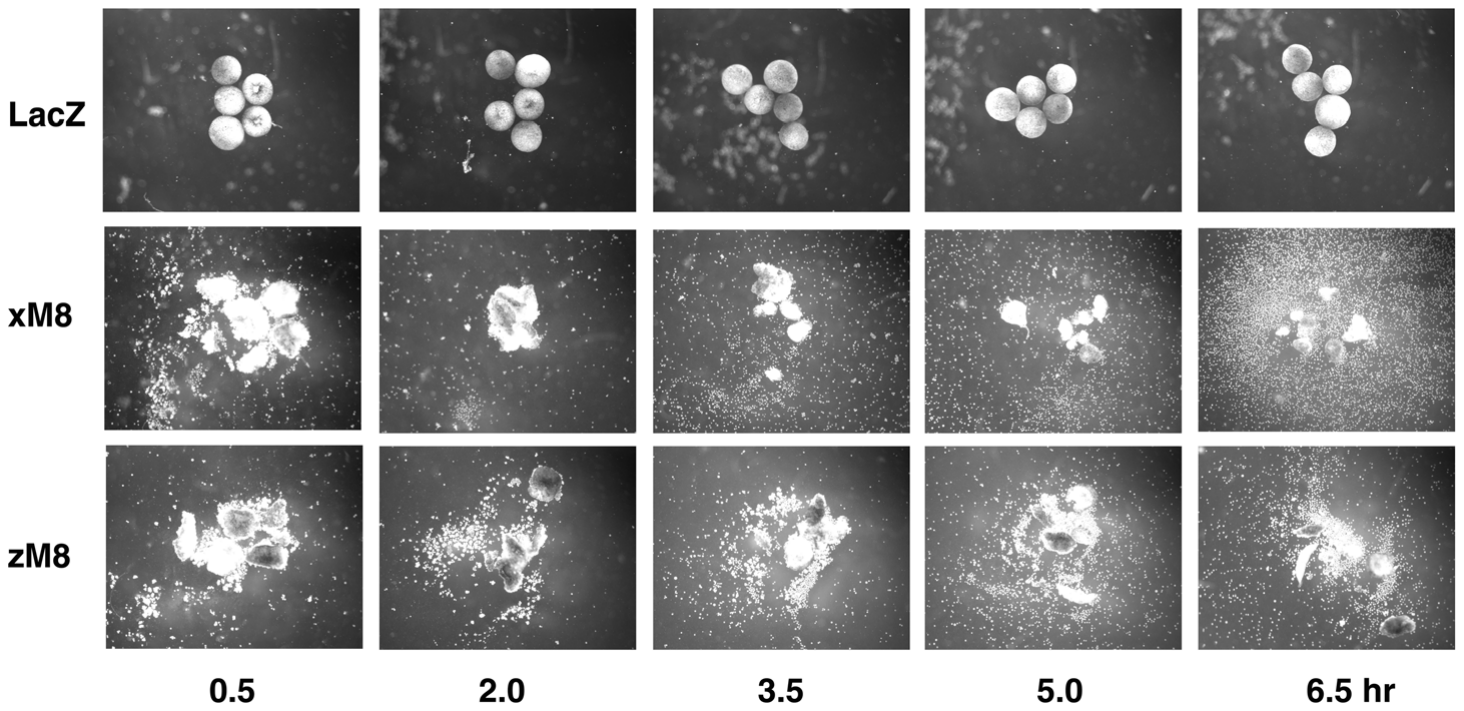
March8 induces cell dissociation in Xenopus animal caps. Xenopus embryos were injected with 400*LacZ*, Xenopus *march8* or zebrafish *march8* mRNA. Animal caps were dissected and incubated as described in Materials and Methods. Caps were photographed at the indicated times after being placed in dissociation media.

*X. laevis* contains a March8 gene highly similar to that in zebrafish and other vertebrates (Figure S1), and this gene is expressed in the early embryo (unpublished observations). Thus we considered it appropriate to use the Xenopus system to test the effect of March8 on cell adhesion. Xenopus embryos injected with *march8* mRNA appeared normal at the late blastula stage when animal caps were dissected, but when the caps were cultured, control caps (LacZ injected) quickly curled up to form a stable ball of cells, whereas *march8*-injected caps shed cells (Figure 4; a similar experiment at a lower injection dose is shown in Figure S4). Both Xenopus and zebrafish March8 had a similar effect. It appeared that the dissociated cells derive from the inner layer of the ectoderm, whereas the outer pigmented layer stayed largely intact. We conclude that overexpression of March8 reduces adhesion between embryonic cells in the Xenopus animal cap.

### March8 down-regulates cell surface levels of E-cadherin

**Figure 5:**
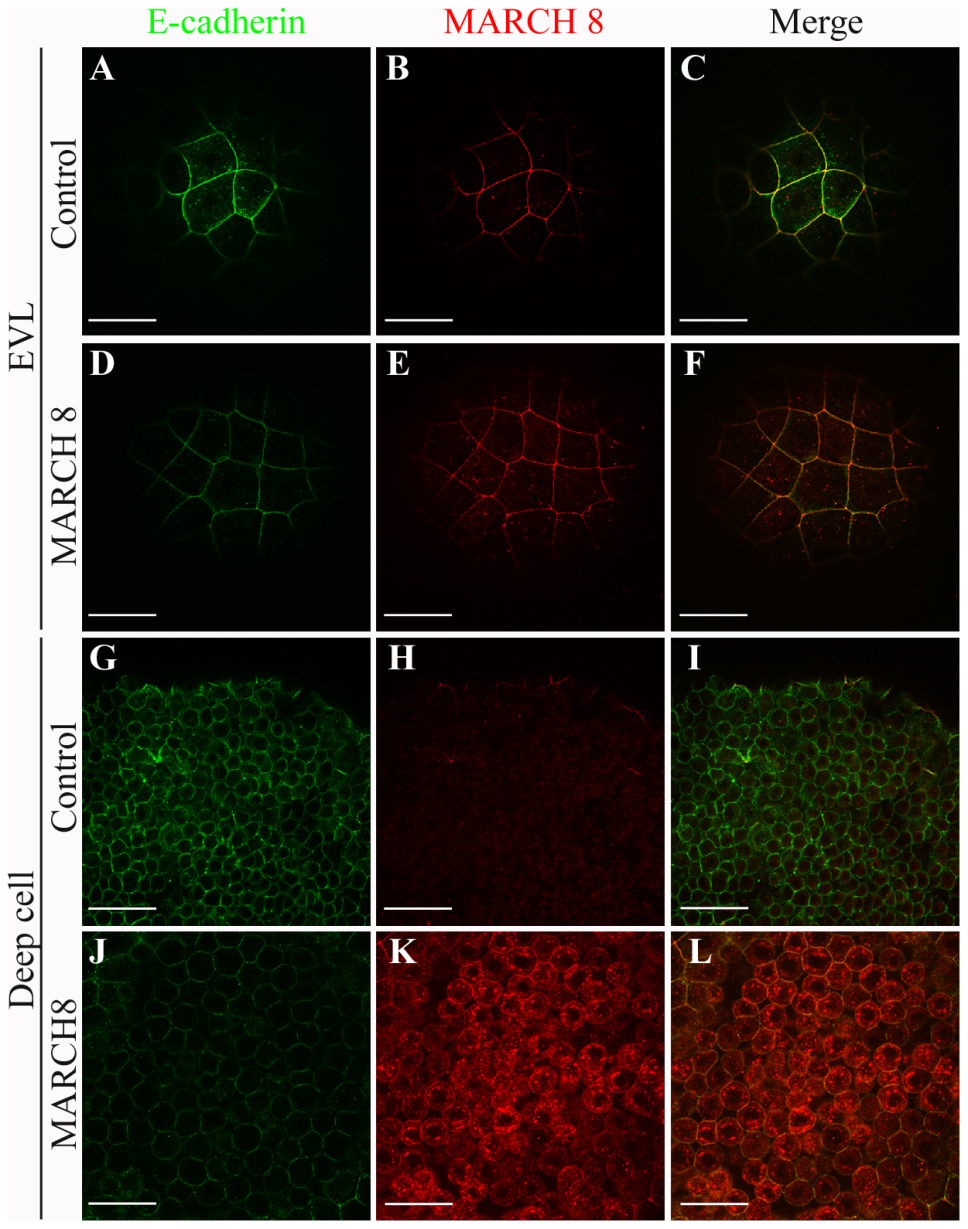
March8 down-regulates cell surface levels of E-cadherin. Control and *march8* mRNA (25 pg) injected embryos were fixed at germ ring stage and stained with anti-March8 (red) and anti-E-cadherin (green) antibodies. Confocal images of EVL and deep cells are shown in animal pole view. March8 overexpression is visualized by higher levels of March8 in intracellular vesicles, and leads to reduced cell surface localization of E-cadherin.

As E-cadherin, a maternally expressed protein, is the major adhesion molecule in zebrafish embryos [4], [5], [6], we asked whether the reduction in cell adhesion resulting from overexpression of March8 is correlated with changes in E-cadherin levels or localization. Zebrafish embryos injected with *march8* mRNA and controls were examined by whole-mount immunofluorescence staining to detect March8 and E-cadherin localization. March8 is expressed during blastula and gastrula stages mostly at the plasma membrane and also in cytosolic vesicles, both in the EVL (enveloping layer) and in deep cells. March8 overexpression increased staining especially in cytosolic vesicles, most notably in deep cells (Figure 5B,E,H,K). E-cadherin was found primarily at the cell surface in control and March8 overexpressing embryos, but the level of surface E-cadherin in EVL and deep cells was reduced after March8 overexpression (Figure 5A,D,G,J). These observations suggest that March8 acts to remove E-cadherin from the cell membrane. March8 has been shown to mediate endocytosis and degradation of various cell surface molecule in different systems [25], [26], [27].

**Figure 6:**
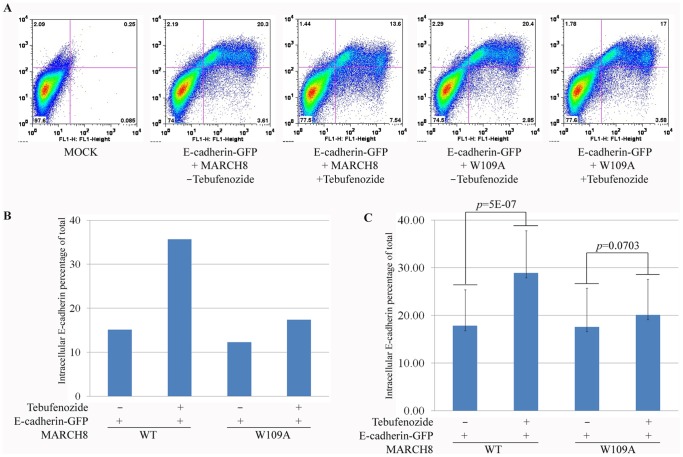
March8 overexpression reduces cell surface expression of E-cadherin in cultured cells. (A) HEK293T cells were transfected with plasmids encoding E-cadherin-GFP, and inducible March8 WT or W109A mutant. After 12 h, cells were treated for 8 h with tebufenozide to induce March8 expression or maintained without the drug, as indicated (see Figure 7 for induction efficiency). For membrane E-cadherin staining, non-permeablized cells were stained using an antibody to an extracellular epitope (red). Cells were analyzed by flow cytometry in red (Y axis; surface E-cadherin) and green channels (X axis; total E-cadherin). Thus the upper right quadrant represents cells with E-cadherin at the surface, while the lower right quadrant represents non-surface (intracellular) E-cadherin. (B, C) Fraction of total E-cadherin that is intracellular. The data from the experiment in (A) are shown in (B). Panel (C) shows the average intracellular E-cadherin measured in 20 separate flow cytometry experiments. Induction of WT March8 led to an increase in intracellular E-cadherin that is highly significant, whereas induction of the W109A mutant did not lead to a significant change (student's T test).

**Figure 7:**
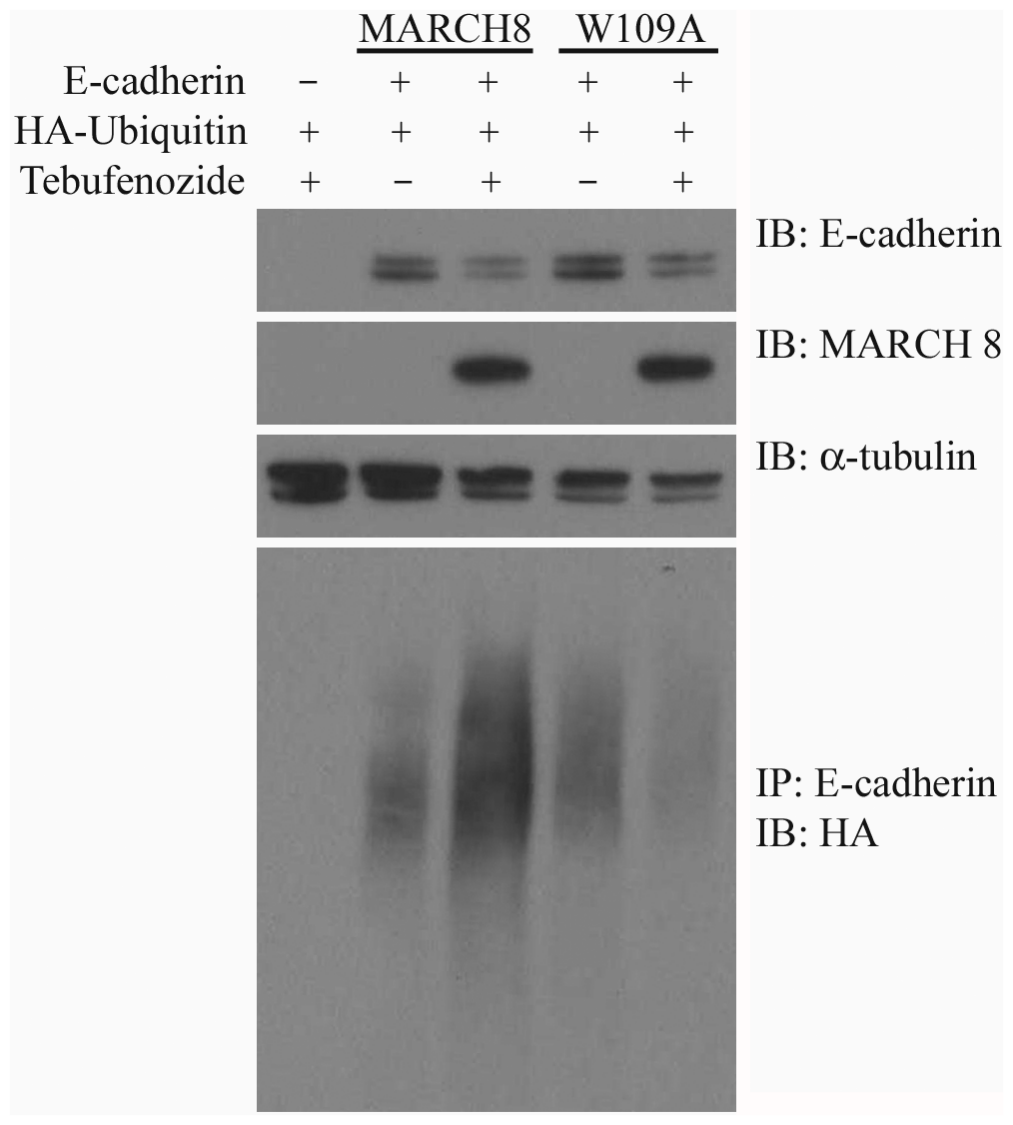
E-cadherin is ubiquitinated by March 8. HEK293T cells were transfected with HA-Ubiquitin, E-cadherin-GFP, and WT or W109A mutant March8, as indicated, and March8 expression was induced with tebufenozide (see Materials and Methods). E-cadherin was immunoprecipitated (IP) from lysates and immunoblotted (IB) with anti-HA antibody to detect ubiquitinated E-cadherin. Expression of the different proteins was checked by IB of lysates. Three additional independent experiments, and quantification of E-cadherin ubiquitination, are shown in Figure S5.

For a quantitative evaluation of the effect of March8 on cell surface E-cadherin we turned to cultured cells to measure the changes in membrane localization by flow cytometry. HEK293T cells were transfected with expression constructs for E-cadherin-GFP and inducible March8. The dual expression construct we used constitutively expresses Gal4-VP16-EcR fusion protein, which is inactive in the absence of an ecdysone agonist [29]. Addition of the agonist tebufenozide results in Gal4-VP16 activator function, leading to the expression of March8 from its UAS-driven module that is part of the same plasmid (see Materials and Methods). Transfected 293T cells with or without tebufenozide induction were stained for extracellular E-cadherin and subjected to FACS analysis in which the red channel counted surface E-cadherin while the green channel counted GFP representing total E-cadherin. A representative experiment is shown in Figure 6A,B. Control cells show no staining in either channel as 293T cells express very low levels of E-cadherin. After transfection but without tebufenozide, most of the cells are in the upper right quadrant, representing E-cadherin localized at the surface and accessible to extracellular staining, and only 15% were in the lower right quadrant representing intracellular E-cadherin (Figure 6A). After induction of March8 the level of staining in the lower right quadrant increased substantially to 36%, representing intracellular E-cadherin. E3 ubiquitin ligase activity was required for this effect, as shown by the fact that transfection of a March8 RING domain mutation (W109A), which abolishes its activity (Figure 2D), has little effect on E-cadherin localization (Figure 6A). Figure 6B shows the quantification of the experiment in Figure 6A. The induction of March8 is illustrated below (Figure 7). The conclusions derived from the experiment shown in Figure 6A,B were substantiated by quantification of multiple independent experiments, providing statistical support for the conclusion that WT but not W109A mutant March8 could downregulate E-cadherin cell surface levels (Figure 6C). Thus, the ligase activity of March8 is essential for E-cadherin downregulation, suggesting that this surface molecule can be ubiquitinated by March8.

Direct assays for E-cadherin ubiquitination support a role for March8 in the process (Figure 7, Figure S5). After transfection into 293T cells, March8 WT but not the W109A mutant mediates the ubiquitination of E-cadherin as seen by immunoblotting with anti-HA after immunoprecipitation of E-cadherin. The gel images and quantification in Figure S5 illustrate that, while recovery of some of the proteins varied, the increase in E-cadherin ubiquitination by March8 induction was reproducible in multiple experiments. These figures also illustrate the tight regulation of March8 expression by the ecdysone receptor/tebufenozide system. We suggest that ubiquitination by March8 is involved in the removal of E-cadherin from the cell surface, regulating cell adhesion in the embryo.

## Discussion

In this study we have explored the possible function of March8 in the early embryo of the zebrafish. The immune system focus of almost all previous studies on March family members may derive in part from the fact that c-MIR, as March8 was named originally, was identified as the cellular homolog of viral proteins that downregulate the immune response [18]. Eleven members have been identified in the March family, and most of them have been found to function in the regulation of the immune system [19], [20]. These functions involve the downregulation of multiple cell surface components including MHC I and MHC II proteins and additional examples [18], [21], [22], [23], [24], [25], [27], [30], [31], [32], [33]. March8 and the closely related March1, which have been studied perhaps more extensively than other family members, act by ubiquitination-dependent endocytosis of target proteins such as MHC II, CD86 (B7.2), TRAIL (TNF-related Apoptosis Inducing Ligand) receptor, transferrin receptor, and others, leading to their degradation by, at least in part, a lysosomal mechanism [18], [22], [23], [24], [26], [27], [30]. March8 may achieve some of these effects by redirecting the traffic of cargo molecules from recycling to lysosomes [34].

The immune system focus of March protein function is generally supported by literature reference to the preferential expression of several family members in immune cells. Bartee and colleagues [21] checked expression of six March proteins using a panel of human tissues. Close inspection of the data show that some March proteins, most notably March1, are indeed most highly expressed in lymph nodes and spleen, although expression in other tissues is also detected. However, other March family members do not show any pronounced preference and are expressed at similar levels in many tissues. Of course, most adult tissue samples include lymphocytes, accounting for some of these observations. Our finding that zebrafish *march8* is expressed in maternal RNA and throughout somitogenesis, long before lymphocytes arise in the embryo [35], shows clearly that March factor expression is widespread. Without questioning the view that March proteins are particularly important in immune system regulation, these observations strengthen the likelihood that these proteins also affect the development and function of other cell types. Consistent with this suggestion, previous work has indicated that March7 and March11, distantly related to March8, function in the differentiation of spermatids [36], [37]. Our experiments add to the available knowledge about non-immune system functions of March proteins in that we show that March8 is required for normal embryogenesis in zebrafish. While we have no mechanistic explanation for the MO-induced phenotype we consider it to be a specific consequence of March8 inhibition because of the ability to largely rescue the embryos with injected MO-resistant March8 mRNA.

The observed cell dissociation in response to excess March8 focused our attention on cadherins, major cell-cell adhesion proteins that have been well studied in Xenopus and zebrafish embryos [3], [14], [38], [39]. Xenopus [9], [10], [11], [12], and zebrafish [4], [5], [6], [7] cadherins are expressed at high levels in the egg and early embryo, and are essential for cleavage, epiboly and gastrulation movements [8], [40], [41]. The essential function of E-cadherin in zebrafish epiboly is most clearly demonstrated in the *half baked* mutant, which affects the *cdh1* locus [6]. Regulation of cadherin function is thus necessary for development, but due to the maternal expression of E-cadherin such regulation is expected to involve posttranscriptional, and likely posttranslational, mechanisms. It has been shown that E-cadherin activity can be regulated by Galpha12/13 binding to its intracellular domain, so that changes in Galpha12/13 action lead to cell dissociation and abnormal cell movements [8]. Further, appropriate intracellular localization of E-cadherin is essential: mutant embryos lacking transcription factor Pou5f1 show delayed gastrulation movements due to misregulation of E-cadherin endosomal trafficking, mediated by EGF regulation of p120 [42]. E-cadherin is a substrate for ubiquitination by the RING class E3 ubiquitin ligase Hakai, which leads to its endocytosis in epithelial cells [17]. The possible function of Hakai in vertebrate embryos is unknown, but the Drosophila homolog is required for normal development in the fly [43]. We found that March8 can lead to removal of E-cadherin from the cell surface, both in embryos and in cultured cells. In the context summarized above these findings suggest that regulating E-cadherin localization and abundance in the embryo is a biological role of March8, thereby making a contribution to the appropriate balance between cell-cell adhesion required for stability and relaxation of adhesion required for cell motility, both of which are indispensible for development.

## Materials and Methods

This research has been approved by the Eunice Kennedy Shriver National Institute of Child Health and Human Development, Bethesda, MD, USA Animal Care and Use Committee.

### RNA extraction and RT-PCR

Total RNA from staged embryos was extracted, and cDNA synthesized.

Primers sequences are as follows:

zMarch8-S-RT; 5′-ATCCCTCGTGATGTCACTTCCTC

zMarch8-A-RT; 5′-CGGTCATCTGAAGCTTCTCCCACTTG

β-actin-S: 5′-GAGGAGCACCCCGTCCTGCTCAC

β-actin-AS: 5′-GATGGCTGGAACAGGGCCTCTG

### mRNA synthesis and microinjection

DNA fragments encoding the open reading frame of zebrafish WT and Ring domain mutant (W109A) *march8* and Xenopus *march8* were inserted into the expression vector pCS2+ and linearized with NotI. RNA was synthesized using the mMESSAGE mMACHINE (Ambion), according to the manufacturer's protocols. RNAs were injected into the yolk of zebrafish embryos at the 1–8 cell stage, and into the animal hemisphere of *Xenopus* embryos at the 2-cell stage.

### Antisense morpholino injection

Antisense 5′UTR *March8* morpholino (MO) was designed and synthesized by GeneTools (Corvallis, OR): 5′- AGCTCACACTCTCTGAGGCTGAAGT-3′ (March8 MO). Ten ng of *March8* MO was injected into the yolk of one cell stage embryos. As the synthetic mRNA we used for injection does not contain UTR sequences (see above) it is resistant to MO inhibition.

### Whole-mount in situ hybridization

The pCS2+- March8 plasmid was linearized with BamHI and antisense digoxigenin (DIG)- labeled probe was synthesized with T7 RNA polymerase following the manufacturer's protocols (Roche). In situ hybridization was done as described [44].

### TUNEL assay

Terminal deoxynucleotidyl transferase dUTP Nick End Labeling (TUNEL) assay was performed using terminal deoxynucleotidyl transferase (TdT) and digoxigenin (DIG)–dUTP in TdT buffer (Roche) following the manufacturer's protocols.

### Animal cap assay

Animal caps were dissected from control and *march8* mRNA-injected *X. laevis* embryos (see above) at stage 9–9.5. Animal caps were incubated in a low salt solution (20 mM NaCl, 2 mM KCl, 2.4 mM NaHCO_3_, 5 mM HEPES pH 7.8, 60 µM CaCl_2_, 30 µM MgSO_4_) with gentle swirling at room temperature. Photographs were taken over the course of several hours to assess cell dissociation.

### Plasmid construction

To construct inducible zebrafish WT or enzymatically inactive RING domain mutant W109A *March8*, we used the Gal4-vp16-EcR (GV-EcR F′)/UAS system which uses a fusion to the insect-specific ecdysone receptor (EcR) to allow small-molecule inducible gene expression [29]. The plasmids were assembled using the multisite Gateway-based construction kit [45]. The entry clones pDONRP4-P1R-CMV:GV-EcR F′, pDONR221-SV40:PUR::5XUAS and pDONRP2R-P3-March8 or W109A were constructed from the components in the two references above, the SV40:PUR module from the PUR vector of Clonetech, and the *march8* coding sequence. The resulting assembled constructs, pDestTol2pA-CMV:GV-EcR F′::SV40:PUR::5XUAS:March8-SV40polyA and pDestTol2pA -CMV: GV-EcR F′:: SV40:PUR::5XUAS:W109A-SV40polyA, contain both components of the dual expression system in the same plasmid. The CMV promoter drives expression of Gal4-Vp16-EcR fusion protein, which is active only after addition of ecdysone agonist. In the responder module, five tandem repeats of the Gal4-recognition sequence (5XUAS) drive transcription of the target gene. In our case, the targets March8 and its W109A mutant are induced by treatment with 50 µM tebufenozide, a nonsteroidal ecdysone agonist [29]. pcDNA3.1-E-cadherin-GFP was from Addgene (plasmid28009). Membrane GFP is a construct containing the CAAX motif, directing GFP to the membrane [46].

### Cell culture and transfection

Human embryonic kidney 293T cells were grown in Dulbecco's Modified Eagle's Medium supplemented with 10% fetal bovine serum and antibiotics at 37°C in an atmosphere containing 5% CO_2_. For transfection, 5×10^5^ cells were plated on 6-well plates for 24 h prior to use, and 700 ng of each plasmid encoding human E-cadherin-GFP, HA-Ubiquitin, and inducible zebrafish WT or W109A mutant *march8* were added. X-tremeGENE HP Transfection Reagent (Roche) was added and processed according to the manufacturer's protocol; membrane-GFP plasmid was added to equalize total DNA.

In ubiquitination assays, target proteins were expressed together with HA-tagged ubiquitin in 293T cells. Lysates were subjected to immunoprecipitation for the target protein, and the isolated complex blotted or HA. For March8 self-ubiquitination assay, 293T cells were incubated for 12 h after transfection and were treated for 8 h with 50 µM tebufenozide to induce March8 expression. For E-cadherin ubiquitination assay, 293T cells were treated for 8 h with 50 µM tebufenozide for March8 induction, and 10 nM Bafilomycin A1 for accumulation of ubiquitinated-E-cadherin [26], [47].

### Immunoblotting and immunoprecipitation

Transfected HEK293T cells were washed in cold PBS, extracted with lysis buffer [10 mM Tris (pH 8.0), 150 mM NaCl, 1 mM DTT, 10% Glycerol, 0.2% NP-40], proteins (20 µg per lane) were separated by NUPAGE 4–12% Bis-Tris Gel (Invitrogen), and transferred to polyvinylidene fluoride membranes (PVDF). The membranes were blocked in TBS-Tween 20 (0.1%, V/V) containing 5% nonfat dry milk, incubated with the primary antibodies in the blocking solution overnight at 4°C, and washed with TBS-T three times for 10 min. Horseradish peroxidase-conjugated secondary antibodies (Cell Signaling Technology) were used to detect the immunoreactive proteins by chemiluminescence. For immunoprecipitation, cell lysates were incubated with the indicated antibody and protein G-sepharose beads (Roche) overnight at 4°C. Beads were washed 10 times in lysis buffer, and proteins were recovered by boiling in SDS sample buffer and analyzed using immnunoblotting. Anti-HA antibody was from Roche, and E-cadherin antibody was from BD Transduction Laboratories. Anti-α-tubulin antibody (Calbiochem) was used as a loading control. A synthetic peptide (CKIKVFDKPSLLEPNLEKEA) derived from zebrafish March8 C-terminal region was synthesized, conjugated to KLH, and used to immunize specific pathogen free (SPF) rabbits (Covance). All these antibodies were used at 1∶5000 dilution.

### Whole-mount immunostaining

Germ ring stage zebrafish embryos were fixed with 20% DMSO/80% Methanol at −20°C overnight. After dechorionation, embryos were blocked at room temperature for 2 h in blocking solution (2% BSA, 0.1% TritonX-100 with 10% Goat serum in PBS). All washes were performed using PBS0.1T (2% BSA and 0.1% TritonX-100 in PBS). Embryos were stained with Anti-E-cadherin (1∶500, BD Transduction Laboratories) and Anti-March8 (1∶500, Proteintech) at 4°C overnight. Secondary antibodies (Alexa Fluor 488 and 568; Molecular Probe) were used at a dilution of 1∶1000. Images were obtained using a Leica TCS SP5 II confocal microscope with a 40× PlanApo objective.

### Flow cytometry

HEK293T cells were plated on 6 well plates for 24 h prior to use. Human E-cadherin-GFP and inducible zebrafish WT or mutant *march8* plasmids were transfected into the cells (see above). Cells were incubated for 12 h and then transferred to fresh media containing 50 µM tebufenozide for 8 h. After incubation, the cells were harvested and non-permeablized cells were stained at 4°C with extracellular domain-specific E-cadherin antibody (67A4, Santa Cruz, 1∶100) for 30 min in FACS buffer (0.5% BSA, 1% Sodium Azide in PBS). Cells were washed 3 times with FACS buffer and were stained with Alexa-647 (1∶1000, Molecular Probe) secondary antibody at 4°C for 30 min. Data acquisition was performed on a FACScan flow cytometer (BD Bioscience), and data were analyzed using FloJo software.

## Supporting Information

Figure S1 **Multiple sequence alignment of March8.** An alignment prepared using Clustal of March8 from diverse species. The conserved RING-CH domain is indicated in red, two transmembrane domains in green, and the conserved tyrosine-based YXXΦ motif in light blue.
(TIF)

Figure S2 **Expression pattern of** ***march8*** **during embryogenesis.** (A) Total RNA was extracted from staged embryos, and *march8* mRNA was analyzed by RT-PCR; β-actin was used as a loading control. (B) *March8* mRNA expression in zebrafish embryos was detected by *in situ* hybridization. Shown are 2 cell, 70% epiboly and tailbud stage; lateral views with animal pole on top. Three and 10 somite; left: lateral view with dorsal to the right; right: dorsal view anterior to the top. One and two day embryos; lateral view with anterior to the left.
(TIF)

Figure S3 **March8 MO injection increases apoptosis in zebrafish embryos.** Detection of apoptotic cells by TUNEL assay in zebrafish embryos at 26 hpf (lateral views). Uninjected embryos showed a low level of apoptotic cells. March8 MO injection increased apoptotic cells in the entire embryo.
(TIF)

Figure S4 **March8 induces cell dissociation in Xenopus animal caps.** Xenopus embryos were injected with 200 pg of *LacZ*, Xenopus *march8* or zebrafish *march8* mRNA. Animal caps were dissected and incubated as described in Materials and Methods. Caps were photographed at the indicated times after being placed in dissociation media.
(TIF)

Figure S5 **March8 mediates ubiquitination of E-cadherin.** Three ubiquitination experiments are shown at the top (A,B,C), complementing Figure 7 in the main text which represents experiment 2 in this series. In all cases 293T cells were transfected with the plasmids indicated, and tebufenozide was added as indicated to induce March8 expression (see Materials and Methods in the main text). Co-immunoprecipitation of E-cadherin and March8 is illustrated in panel A. To assay ubiquitination, E-cadherin was immunoprecipitated, and the isolated complex blotted for ubiquitin by way of its HA epitope tag. All experiments show an increase in ubiquitinated E-cadherin after induction of wild type but not mutant W109A March8. The four experiments, including the one shown in Figure 7, were quantified by Image J. The ratios of HA-ubiquitinated-E-cadherin to tubulin in the different lanes are plotted in the histogram below (D), where the lane designations correspond to those in the gel images. The relevant comparisons are between lane 2 (March8 not induced) and lane 3 (March8 induced). Ubiquitination after induction of WT March8 increased in each of the four experiments. The average film density (arbitrary units) in lane 2 was 3.6, and 9.0 in lane 3 as shown on the right (E), where the standard deviations and the p value (student's T test) are also given. The difference between not induced and induced values is highly significant.
(TIF)
