# Supplementary figures and images for: Cell Adhesion in Zebrafish Embryos Is Modulated by March8
Source: PLoS One. 2014 Apr 21;9(4):e94873. doi: 10.1371/journal.pone.0094873 (PMC3994051; doi:10.1371/journal.pone.0094873)

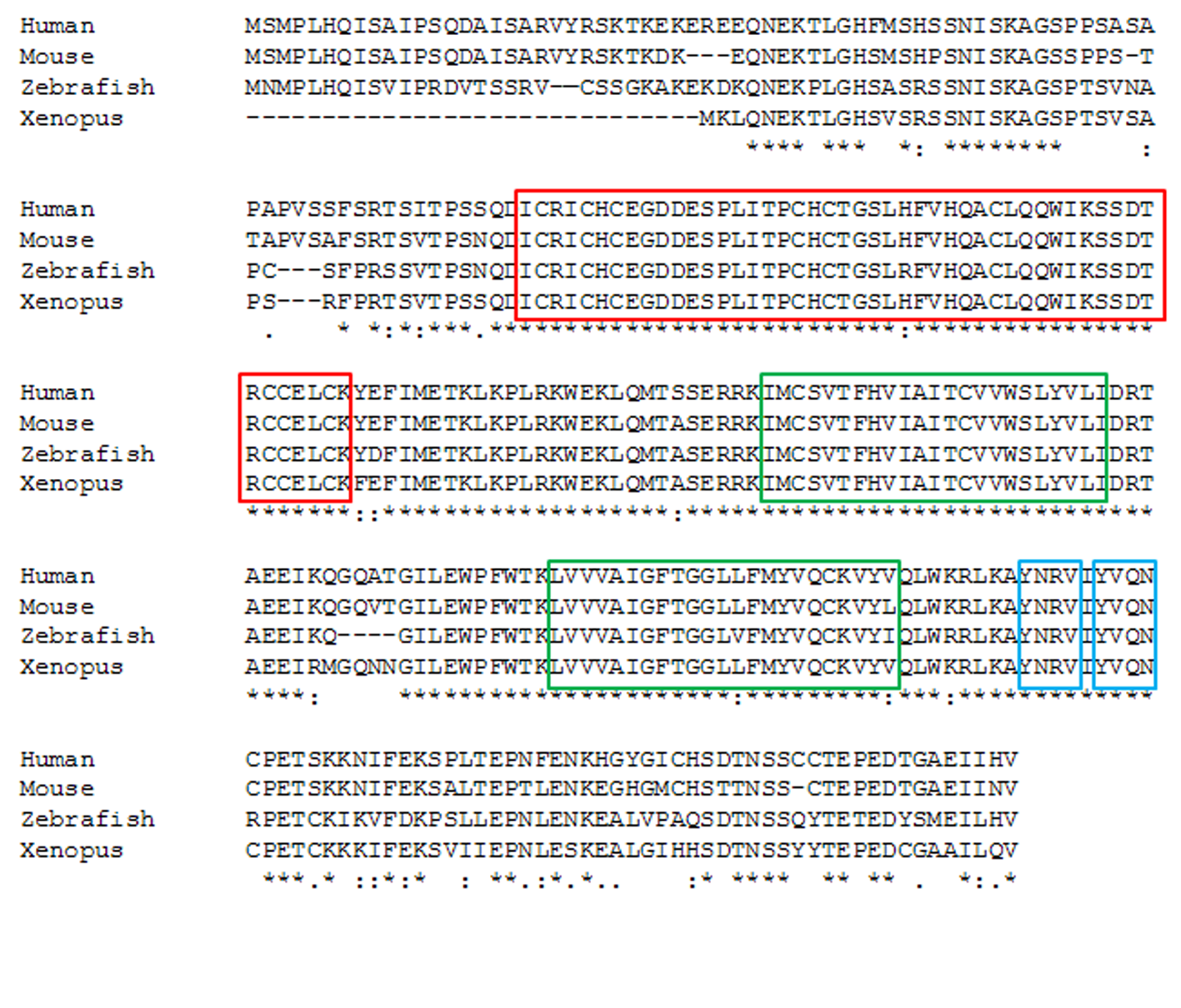

Supplement: Figure S1 — Multiple sequence alignment of March8. An alignment prepared using Clustal of March8 from diverse species. The conserved RING-CH domain is indicated in red, two transmembrane domains in green, and the conserved tyrosine-based YXXΦ motif in light blue. (TIF) [file pone.0094873.s001.tif]

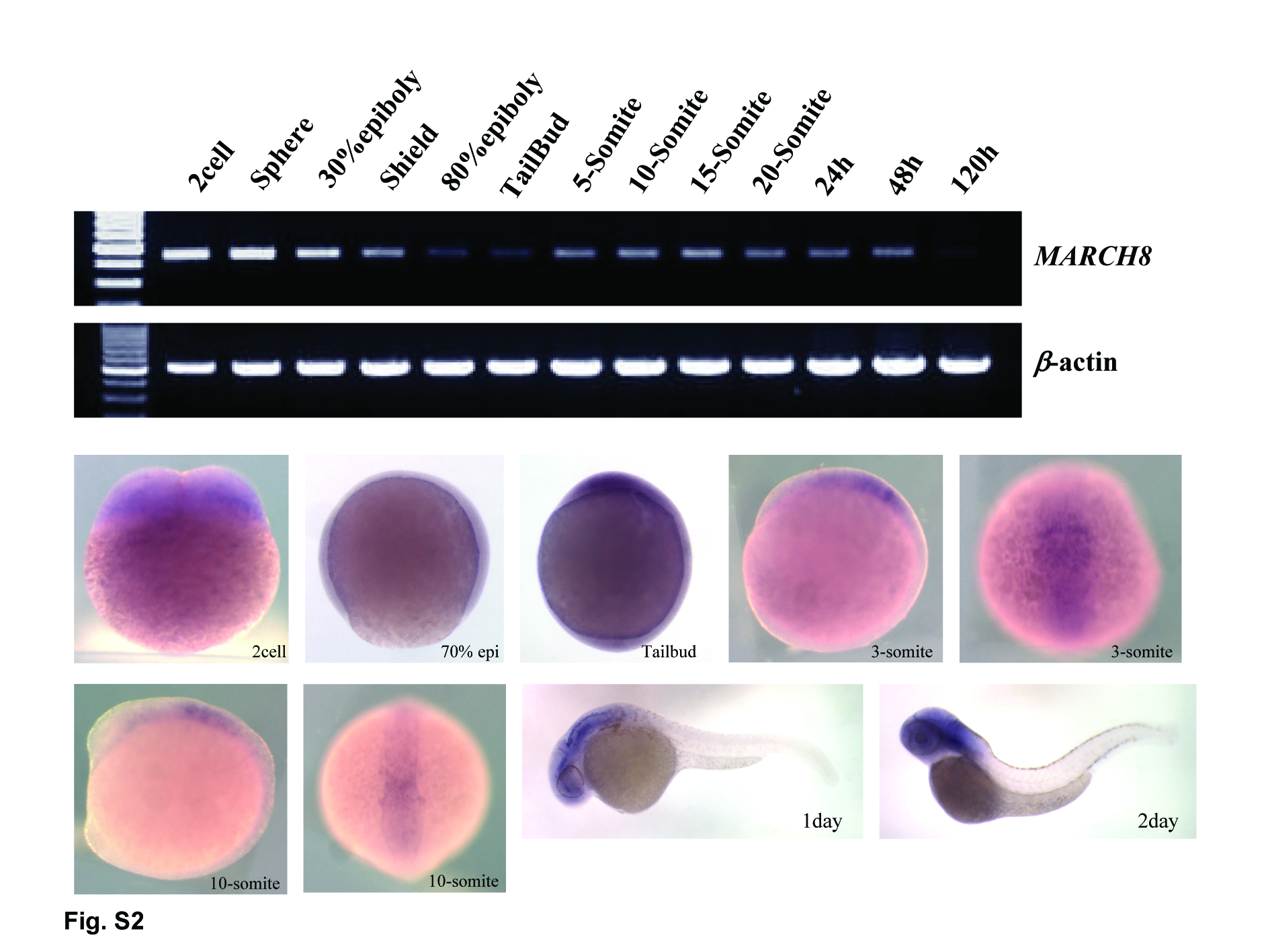

Supplement: Figure S2 — Expression pattern of march8 during embryogenesis. (A) Total RNA was extracted from staged embryos, and march8 mRNA was analyzed by RT-PCR; β-actin was used as a loading control. (B) March8 mRNA expression in zebrafish embryos was detected by in situ hybridization. Shown are 2 cell, 70% epiboly and tailbud stage; lateral views with animal pole on top. Three and 10 somite; left: lateral view with dorsal to the right; right: dorsal view anterior to the top. One and two day embryos; lateral view with anterior to the left. (TIF) [file pone.0094873.s002.tif]

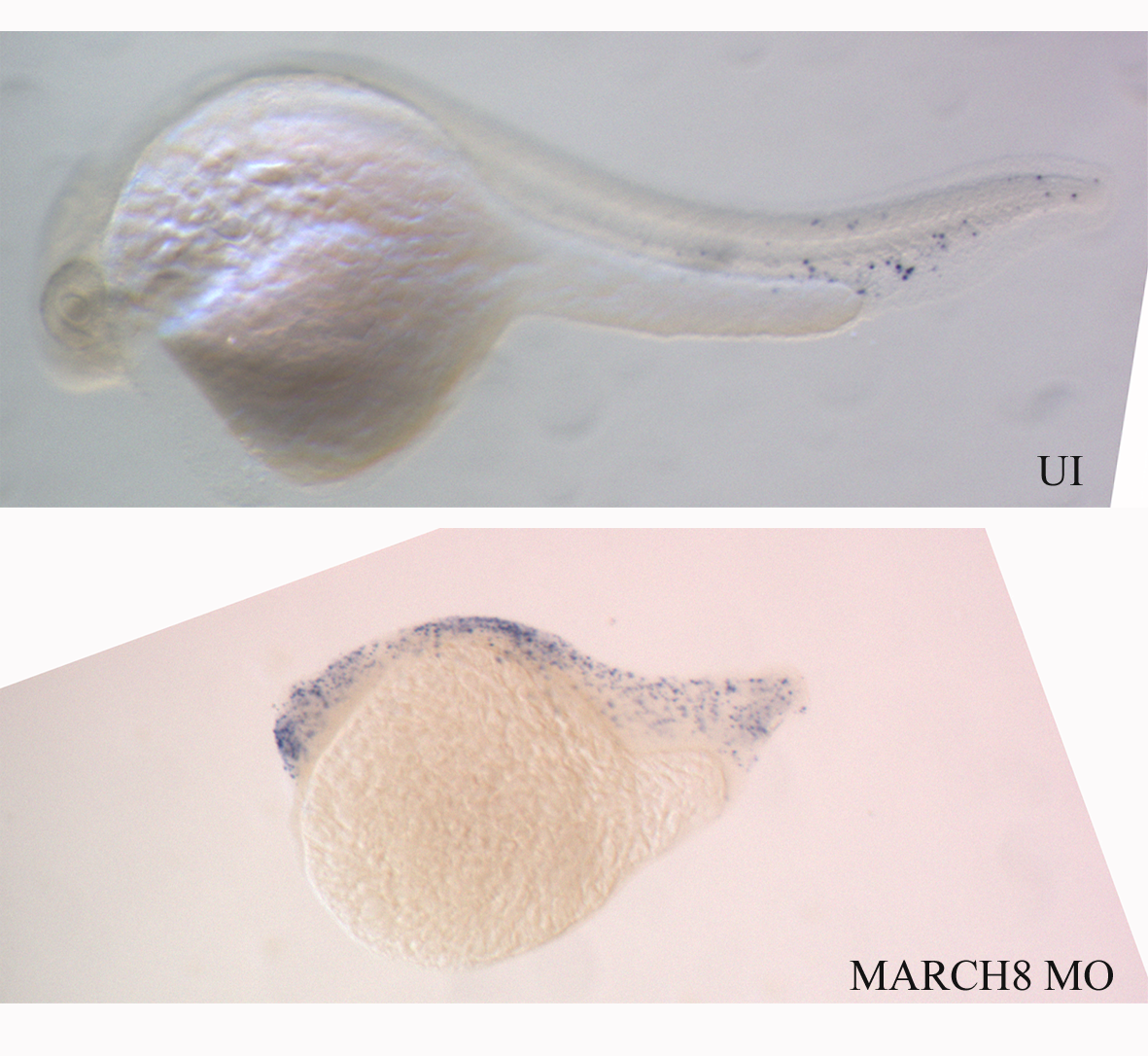

Supplement: Figure S3 — March8 MO injection increases apoptosis in zebrafish embryos. Detection of apoptotic cells by TUNEL assay in zebrafish embryos at 26 hpf (lateral views). Uninjected embryos showed a low level of apoptotic cells. March8 MO injection increased apoptotic cells in the entire embryo. (TIF) [file pone.0094873.s003.tif]

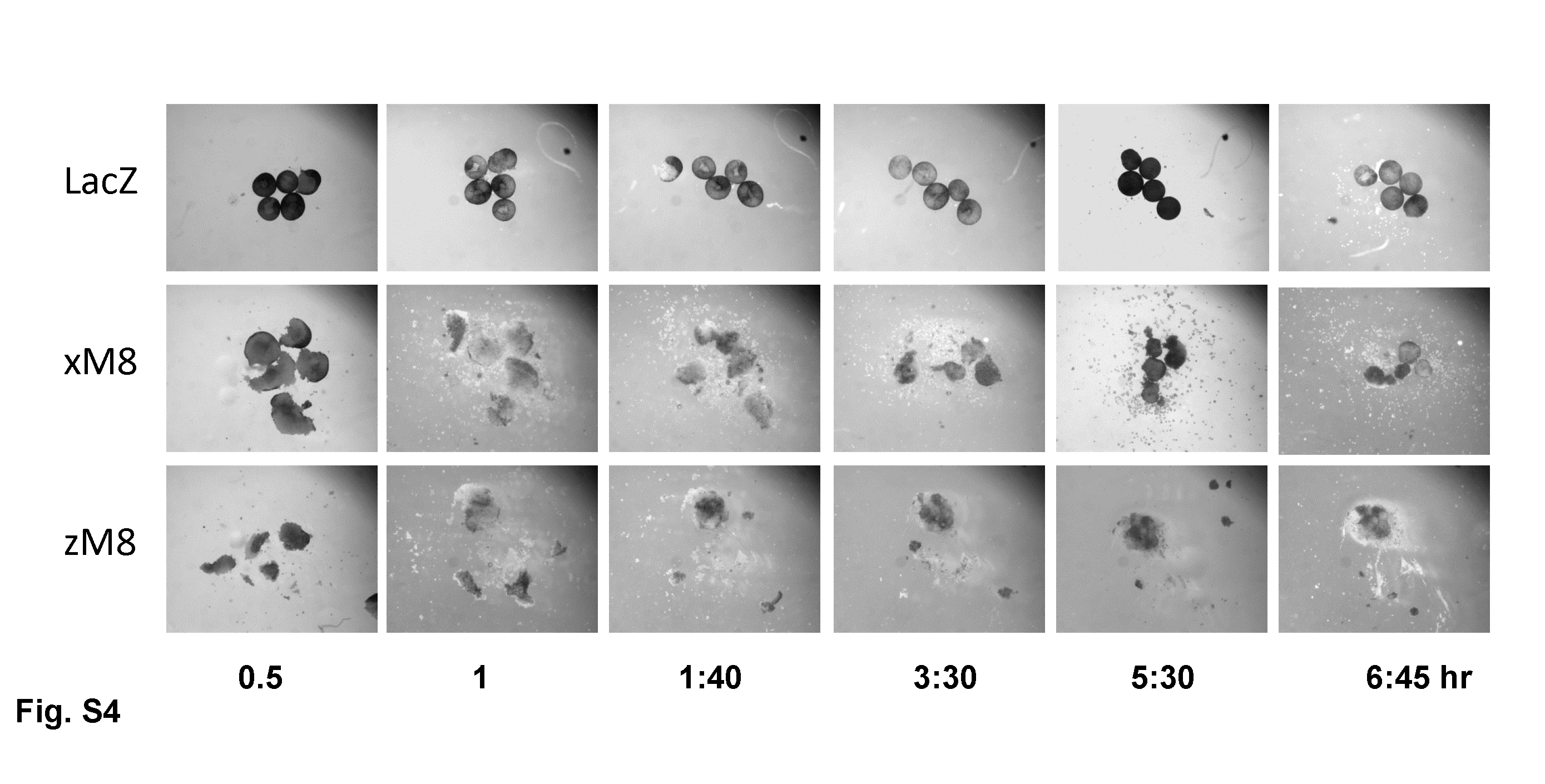

Supplement: Figure S4 — March8 induces cell dissociation in Xenopus animal caps. Xenopus embryos were injected with 200 pg of LacZ, Xenopus march8 or zebrafish march8 mRNA. Animal caps were dissected and incubated as described in Materials and Methods. Caps were photographed at the indicated times after being placed in dissociation media. (TIF) [file pone.0094873.s004.tif]

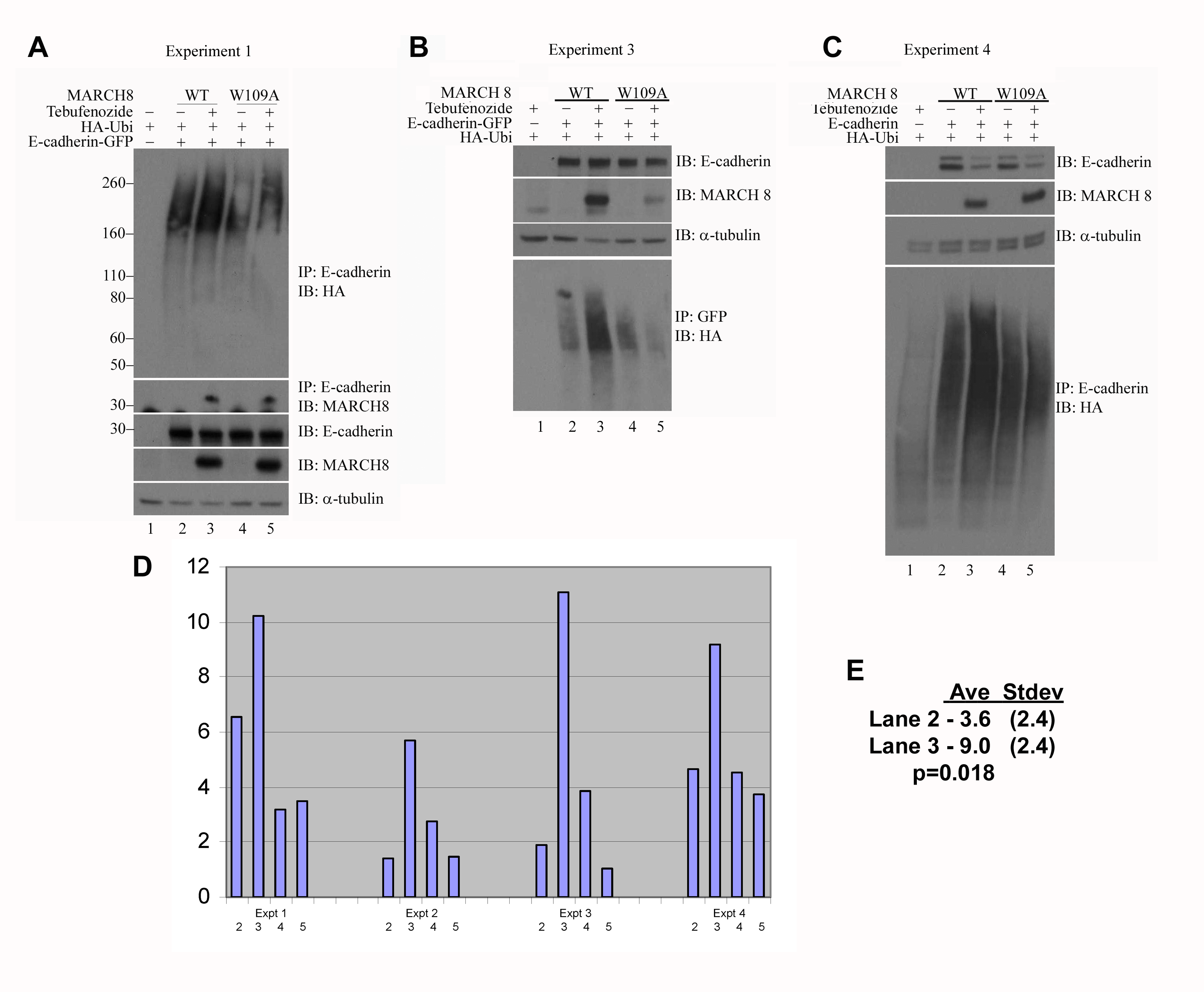

Supplement: Figure S5 — March8 mediates ubiquitination of E-cadherin. Three ubiquitination experiments are shown at the top (A,B,C), complementing Figure 7 in the main text which represents experiment 2 in this series. In all cases 293T cells were transfected with the plasmids indicated, and tebufenozide was added as indicated to induce March8 expression (see Materials and Methods in the main text). Co-immunoprecipitation of E-cadherin and March8 is illustrated in panel A. To assay ubiquitination, E-cadherin was immunoprecipitated, and the isolated complex blotted for ubiquitin by way of its HA epitope tag. All experiments show an increase in ubiquitinated E-cadherin after induction of wild type but not mutant W109A March8. The four experiments, including the one shown in Figure 7, were quantified by Image J. The ratios of HA-ubiquitinated-E-cadherin to tubulin in the different lanes are plotted in the histogram below (D), where the lane designations correspond to those in the gel images. The relevant comparisons are between lane 2 (March8 not induced) and lane 3 (March8 induced). Ubiquitination after induction of WT March8 increased in each of the four experiments. The average film density (arbitrary units) in lane 2 was 3.6, and 9.0 in lane 3 as shown on the right (E), where the standard deviations and the p value (student's T test) are also given. The difference between not induced and induced values is highly significant. (TIF) [file pone.0094873.s005.tif]
